# Genome editing with CRISPR/Cas9 in *Pinus radiata* (D. Don)

**DOI:** 10.1186/s12870-021-03143-x

**Published:** 2021-08-10

**Authors:** Charleson Poovaiah, Lorelle Phillips, Barbara Geddes, Cathie Reeves, Mathias Sorieul, Glenn Thorlby

**Affiliations:** Scion, Rotorua, 3010 New Zealand

**Keywords:** Genome editing, CRISPR, Somatic embryogenesis, *Pinus radiata*, Conifers

## Abstract

**Background:**

To meet increasing demand for forest-based products and protect natural forests from further deforestation requires increased productivity from planted forests. Genetic improvement of conifers by traditional breeding is time consuming due to the long juvenile phase and genome complexity. Genetic modification (GM) offers the opportunity to make transformational changes in shorter time frames but is challenged by current genetically modified organism (GMO) regulations. Genome editing, which can be used to generate site-specific mutations, offers the opportunity to rapidly implement targeted improvements and is globally regulated in a less restrictive way than GM technologies.

**Results:**

We have demonstrated CRISPR/Cas9 genome editing in *P. radiata* targeting a single-copy cell wall gene GUX1 in somatic embryogenic tissue and produced plantlets from the edited tissue. We generated biallelic INDELs with an efficiency of 15 % using a single gRNA. 12 % of the transgenic embryogenic tissue was edited when two gRNAs were used and deletions of up to 1.3 kb were identified. However, the regenerated plants did not contain large deletions but had single nucleotide insertions at one of the target sites. We assessed the use of CRISPR/Cas9 ribonucleoproteins (RNPs) for their ability to accomplish DNA-free genome editing in *P*. *radiata.* We chose a hybrid approach, with RNPs co-delivered with a plasmid-based selectable marker. A two-gRNA strategy was used which produced an editing efficiency of 33 %, and generated INDELs, including large deletions. Using the RNP approach, deletions found in embryogenic tissue were also present in the plantlets. But, all plants produced using the RNP strategy were monoallelic.

**Conclusions:**

We have demonstrated the generation of biallelic and monoallelic INDELs in the coniferous tree *P. radiata* with the CRISPR/Cas9 system using plasmid expressed Cas9 gRNA and RNPs respectively. This opens the opportunity to apply genome editing in conifers to rapidly modify key traits of interest.

**Supplementary Information:**

The online version contains supplementary material available at 10.1186/s12870-021-03143-x.

## Background

Population growth, coupled with the need to transition from a petrochemical-based economy towards a more sustainable bio-based one, is predicted to increase the demand for wood and other forest-based products three-fold by 2050 [1]. This increased demand, together with the challenges associated with climate change and the need to increase agricultural production, will put further pressure on the area and quality of natural forests. It has been estimated that planted forests, which comprise only 7 % of the global forest area, have the potential to supply two-thirds of global roundwood demand [2] and offer a route to sustainably increase the production of forest products and reduce pressure on natural forests. To meet the increased demand, it will be necessary to further increase the productivity of planted forests.

There is a long history of productivity improvements in commercially planted conifer species through traditional breeding and silviculture [3, 4]. The use of genomic-based breeding technologies, particularly the implementation of genomic selection, are also showing promise for implementation into breeding programs [5–7]. Long breeding cycles, large and complex genomes, variable juvenile-mature correlations, emerging pests and diseases, climate, and market changes provide challenges to breeding approaches that have to date led to moderate gains in conifers [6, 8].

Direct manipulation of conifer genomes offers a potentially more rapid route to trait improvement and allows the introduction of novel traits as well as improvement of existing ones. Demonstrated trait modifications in conifers include; insect resistance [9, 10], herbicide tolerance [11, 12], wood pulping efficiency [13, 14], stress tolerance [15] and sterility [16]. These technologies also enable production of rationally designed trees that produce biochemicals and biomass for specific purposes [17], yet, no modified conifers have been commercialized. These modification technologies require the introduction of new genes either via *Agrobacterium* or biolistic based methods [18]. But, the transformation of conifers is challenging, relying on complex somatic embryogenesis protocols, with many species or genotypes proving recalcitrant to somatic embryogenesis protocols and/or transformation [18]. The lack of efficient transformation systems for elite germplasm intended for large-scale production remains a major challenge for genetically modified varietal forestry [19].

Over the last decade, genome editing, particularly the CRISPR/Cas9 system, has been widely used in plants, both for fundamental research and precision breeding [20–22], with the first genome-edited food introduced into the market in 2019 [23]. Novel traits or traits difficult to achieve by breeding, such as biotic- and abiotic-stress resistance [24–26], and sterility [27] can be generated by knockout-mediated trait improvement. Desirable traits can be fine-tuned by generating a range of alleles through either genome editing or base editing [28–31]. Successful demonstrations of editing have included trees like poplar and eucalyptus [32–35]. As far as we are aware, genome editing is yet to be demonstrated in coniferous trees.

Globally, organisms that have had foreign DNA introduced into their genome are considered to be GMOs and are subject to various levels of regulation. However, genome edited plants where the transgene has been removed by crossing and segregation, are not regulated as GMOs in many countries, including Australia, Argentina, Canada, Japan, and the USA [36, 37]. Yet, the European Union and New Zealand still considers such mutated plants to be GMOs and regulates them accordingly [38].

In conifers, removing transgenes by segregation is challenging due to their long breeding timescales. Genome editing mediated by direct delivery of CRISPR/Cas9 ribonucleoproteins (RNPs), circumvents the introduction of new DNA into the plant genome, and as above would not be regulated as GMOs in many countries [39]. The ability to produce edited plants without the requirement to undergo time consuming breeding to remove transgenes makes the use of RNP-mediated editing particularly attractive for slow-breeding conifers.

*Pinus radiata* D. Don., a conifer species native to California, is the world’s most extensively planted exotic softwood [40] due to its high productivity and suitability for the construction timber, furniture, pulp and paper industries [41]. It is predominantly planted in Australia, Chile, and Spain and is the dominant species in New Zealand planted forests, where it comprises 90 % of the planted production forest area and contributes 1.6 % to GDP [42]. We have investigated the use of CRISPR/Cas9 to edit the *P. radiata* glucuronic acid substitution of the xylan 1 (GUX1) gene [43, 44] and demonstrated genome editing using DNA and RNPs.

## Results

### Genome editing with single guide RNA (gRNA)

**Fig. 1:**
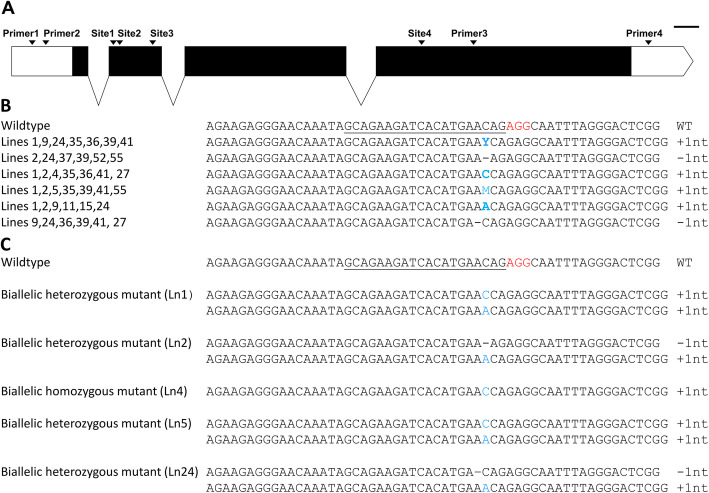
Editing of *GUX1*with a single guide RNA. **A**. Graphical representation of the *GUX1* gene sequence showing the gRNA target sites and primer binding sites. **B**. Pattern of edits identified in the 15 somatic embryogenic tissue lines. **C**. Allelic variations identified in different plantlets. Edited plantlet line numbers are denoted in parenthesis. CRISPR gRNA sites are underlined. Red text denotes the PAM site, blue text denotes insertions and “-“ denotes deletions. ‘Y’ denotes a cytosine or thymine nucleotide. Scale bar 100 bp

We used the cell wall gene GUX1 (NCBI Accession No: MT628352) to investigate genome editing. Bioinformatic analysis (unpublished) has shown that only a single gene encoding the GUX1 protein is present in our in-house *P. radiata* and the publicly available *Pinus taeda* [45] genome sequences. Using a single gRNA at site1 (Fig. 1A), we generated 100 independent transgenic lines by co-transformation with two separate plasmids, one encoding CRISPR/Cas9 and the other, a gRNA and a selectable marker (Fig. S1A, B; Additional file 1). The control lines were transformed with the plasmid containing the gRNA and selectable marker but without the plasmid encoding CRISPR/Cas9. Transgenic embryogenic tissue growing on selective media was screened by Sanger sequencing of PCR products generated using primer1 and primer3 flanking the targeted region (Fig. 1A) to identify edited material. Initial testing identified 15 lines harbouring edits out of the 100 transgenic lines generated. All of the edited lines had a single base pair insertion or deletion at the target cut site (Fig. 1B, Fig. S2A; Additional file 2). Embryogenic tissue containing edits was matured to produce somatic embryos that were germinated to generate *in vitro* plantlets. Of the 15 edited lines, only 2 produced somatic embryos that were able to germinate. A total of 6 plantlets were produced, 5 plantlets germinating from line 1 and one from line 24. DNA was extracted from needle material collected from these plantlets. The PCR products spanning the region of interest were sequenced to confirm edits. All the plantlets, as expected, contained a single base insertion or deletion (Fig. 1C, Fig. S2B; Additional file 2). No edits were found in the lines transformed with gRNA only constructs (Fig. S1A; Additional file 1). To further characterize the gene editing mediated modifications, we cloned the PCR products from the needles of the plantlets into an *E. coli* vector and sequenced 10 colonies derived from each plantlet. All insertions were either an adenine or cytosine base (Fig. 1C). No guanine or thymine insertion was detected in the edited plantlets. All the sequenced plantlets contained biallelic mutations, with one line being homozygous for the insertion (Fig. 1C). Different types of edits in the four edited plants (line 1 to line 5, Fig. 1C) originating from the same embryogenic tissue line was not expected. Line 3 died before analysis could be performed. Based on the *in silico* translated peptide sequence, the single base pair insertion/deletion is predicted to produce a non-functional truncated protein due to premature termination by an in-frame stop codon.

### Genome editing with two gRNAs

**Fig. 2:**
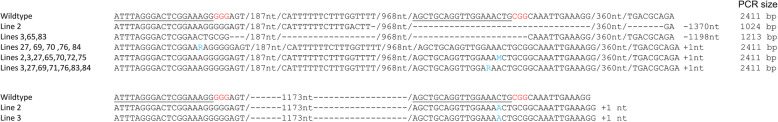
Editing of *GUX1* with two guide RNAs. **A**. Pattern of edits identified in the 12 somatic embryogenic tissue lines. **B**. Details of edits identified in needle tissue from germinated plantlets. CRISPR gRNA sites are underlined. Red font denotes the PAM site and blue font denotes insertions and “-“ denotes deletions. ‘R’ denotes an adenine or guanine nucleotide, ‘M’ denotes an adenine or cytosine nucleotide

A strategy using two gRNAs targeting sites 1.2 kb apart within the GUX1 gene (Fig. 2A) was tested for its ability to produce deletions that could be easily screened by PCR. One hundred transgenic lines were generated. Sequencing and subsequent analysis indicated that 12 lines (12 %) were successfully edited. The control lines were transformed with a plasmid encoding Cas9 and a selectable marker but not the gRNA encoding plasmid (Fig. S2; Additional file 2). Two embryogenic lines with deletions greater than 1 kb were identified (Fig. S3A; Additional file 3). Line 3 had a 1.2 kb deletion at the target cut sites (Fig. 2A, Fig. S2D; Additional file 2), while line 2 had a 1.37 kb deletion, 209 nucleotides downstream of target site 2 (Fig. 2A, Fig. S2C; Additional file 2). Other edited lines had single nucleotide insertions at one of the target sites (Fig. 2A). No edits were found in the 2 lines transformed with Cas9 only construct. When the edited embryogenic tissue was matured to produce embryos, line 2 produced a single plantlet while 4 plantlets were produced from line 3 (Fig. 2B). PCR products, amplified from DNA extracted from needles, were cloned and sequenced from these plantlets. The plantlets did not harbour the 1.37 kb or the 1.2 kb deletions identified in the embryogenic tissue, instead the plantlets had a single insertion at target site 4 and were biallelic (Fig. 2B). Other successfully edited embryogenic lines did not produce plantlets. Like the earlier edits, these plantlets are predicted to introduce a premature stop codon producing a truncated protein.

### Genome editing with ribonucleoprotein (RNP) complexes

**Fig. 3:**
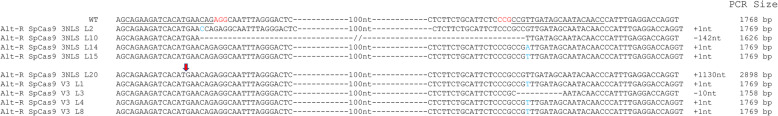
Details of editing in *GUX1* using RNPs. Somatic embryogenic tissue and needles from germinated plantlets showed the same editing pattern. The red arrow shows the location of 1130 bp insertion. CRISPR gRNA sites are underlined and the PAM sequence is denoted by red nucleotides and “-“ denotes deletions

To investigate the use of DNA-free genome editing in *P. radiata*, we tested the ability of directly delivered RNPs, comprising a Cas9 protein in complex with targeting gRNAs, to carry out editing. Because of challenges associated with direct selection of edited plantlets we used a hybrid approach to biolistically co-deliver RNPs with a plasmid-borne selectable marker (Fig. S1E) to select for antibiotic-resistant transformed/edited cells [46]. Two different commercial SpCas9 proteins; Alt-R SpCas9 3NLS and Alt-R SpCas9 V3 were tested for their ability to edit the GUX1 gene. Guide RNAs 142 bp apart (Fig. 3) were used with the intention of generating deletions. Twenty-two antibiotic-resistant transgenic embryogenic lines were generated for Alt-R SpCas9 3NLS and 12 lines for Alt-R SpCas9 V3. Alt-R SpCas9 3NLS produced 5 edited embryogenic lines (22 %) and Alt-R SpCas9 V3 produced 4 edited embryogenic lines (33 %). Embryogenic tissue showed a variety of editing patterns (Fig. 3). Six lines had a single nucleotide insertion at either one of the target sites. The single nucleotides inserted were either adenine, cytosine or thymine. Guanine insertions were not found in our analysis. Alt-R SpCas9 3NLS line 10 had a 142-nucleotide deletion between the target sites. Alt-R SpCas9 3NLS line 20 had a 1130 bp insertion at target site 1 (Fig. 3, Fig. S2E, Fig. S3B; Additional file 2 & 3) while Alt-R SpCas9 V3 line 3 had a 10 bp deletion at target site 3 (Fig. 3, Fig. S2E). The edited embryogenic lines were then matured to produce embryos and subsequently plantlets. Germinated plantlets were then analysed by PCR amplification (Fig. S3B; Additional file 3), cloning and Sanger sequencing. All the resulting plantlets maintained the INDELs seen in their respective embryogenic tissue, including the142-nucleotide deletion in Alt-R SpCas9 3NLS line 10 (Fig. S2E, Fig. S3B; Additional file 2 & 3). Alt-R SpCas9 3NLS line 20 did not produce plantlets (Fig. 3). All embryos/plantlets produced using RNPs were monoallelic.

## Discussion

The CRISPR/Cas9 system is a powerful tool for targeted mutagenesis. It has been widely applied in model species and increasingly in plants of agronomic or horticultural importance [47, 48] and trees [35, 49, 50]. Here, we demonstrate CRISPR/Cas9- mediated genome editing in the commercially important coniferous tree *P. radiata* using both transgenic-mediated production of the editing complex and through the direct delivery of RNPs.

CRISPR/Cas is much simpler to implement than the other reported systems such as ZFN and TALEN [51, 52]. High-efficiency editing can be achieved even in large and complex plant genomes at each of the multiple targeted genomic loci [53, 54]. In this study we demonstrate that this system can efficiently, and site-specifically, edit an endogenous gene in *P. radiata* to produce mono- and bi-allelic mutations.

When editing with a single gRNA using a transgenic approach all edited plantlets had both alleles edited. Production of trees carrying a biallelic edit through crossing or selfing of monoallelic ones would be time consuming. Biallelic edits are desirable for rapid implementation. Targeting the GUX1 gene with a single gRNA produced single nucleotide INDELS. We did not see large deletions using a single gRNA as are frequently seen in other plant species [55–57]. In single gRNA edited plantlets all the insertions were either a cytosine or an adenine. The pattern of insertion or deletion is determined by nucleotide − 4 from the protospacer associated motif (PAM) site [58]. The − 4 nucleotide in the gRNA site1 (Fig. 1A) is an ‘A’, which is predicted to mainly show double-strand break repair via insertion. A repeat of the PAM-distal nucleotide adjacent to the cut site is favoured over other nucleotides resulting, in this case, to be adenine [59]. When editing with a single guide RNA all plants were biallelic (carried edits in both alleles). However, only 20 % of the edited plants that were regenerated carried the same edit on both alleles. Such biallelic edits are desirable for implementation as producing biallelic edits through crossing and segregation of trees carrying monoallelic ones would be time-consuming.

Screening of embryogenic tissue edited with two gRNAs identified large deletions in embryogenic tissue. However, none of the plantlets generated from this material contained these large deletions, instead the plantlets that were produced had only a single nucleotide insertion at one of the two target sites. This may be the result of the embryogenic tissue being chimeric because of delayed Cas9 cleavage occurring in the proliferating embryogenic tissue. This could result in plantlets being generated from differently edited sectors [60, 61]. Similar results have been observed in rice, where genomic deletions were present in transformed callus lines and plants with genomic deletions were rarely found [62]. Furthermore, the rate of genomic deletions with multiple gRNAs is considerably lower than single-site mutagenesis [63], which could have reduced the frequency of plants with large deletions.

It has been suggested that delayed Cas9 cleavage, after the first cell division, in proliferating cells can result in the production of chimeric editing [60, 61]. This may be occurring in the proliferating embryogenic material with plants being generated from embryogenic material derived from different edited cells within the cell line to those identified in the initial screening. This may also explain why embryos carrying different edits were generated from the same embryogenic mass in experiments using a single gRNA.

Genome editing with RNPs has been successfully applied in several species using protoplasts [39, 64–66] and immature embryos [67, 68]. In our study we have successfully edited *P. radiata* somatic embryogenic cells and produced plantlets using RNPs. All RNP edited plantlets were monoallelic, whereas plasmid-mediated CRISPR/Cas9 genome-edited plants were all biallelic. RNPs have a limited time frame for the cas9 protein to cleave DNA to mediate editing before they are degraded by endogenous proteases, resulting in a higher likelihood of monoallelic mutation [69]. All culture and transformation experiments were carried out at 23 °C which is below the optimum for the Cas9 protein activity (37 °C) [70]. This sub-optimum temperature, combined with the short half-life of the RNP complex, may have contributed to the lack of biallelic edits. In contrast to editing using the transgenic delivery of the editing complex, deletions identified in embryogenic tissue were also present in the needles of plantlets produced from this tissue. This is likely also a product of the limited window of activity for editing by RNPs. Edited tissue is likely to be homogenous, as editing would only occur in the cells prior to their first division.

Genome editing efficiency from methods using plasmid DNA and RNPs cannot be compared directly due to the different types of molecules and concentrations used for editing. The efficiency of genome editing using transgenic (12–15 %) and RNP (22–33 %) based delivery of the editing machinery are similar to those using comparable approaches in other plant species [48] and will support the development of efficient editing platforms.

To date gene editing has not been widely applied in tree species. Edited trees have been produced in several perennial fruit trees including, apple [64] and citrus [25, 71] and in the commercially important angiosperm hardwood tree species poplar and eucalyptus [32, 34, 72]. Proof-of-concept demonstrations of editing using protoplasts (without further growth of the edited material) are also available [64, 65] including recently for *Larix gmelinii* (Dahurian larch), a coniferous tree [73]. These examples, particularly demonstrations of editing in protoplasts, suggest that the key challenge to producing gene edited tree species is the lack of robust plant modification methodology. For instance, regeneration of trees from protoplasts of conifer species has resulted in limited success [74, 75] and regeneration of plants from transformed protoplasts of conifers has not been achieved yet. In our study edited plants have been produced from somatic embryogenic cell cultures.

## Conclusions

In this work we have demonstrated genome editing in *P. radiata* using both transgenic and RNP approaches. As far as we are aware, this is the first published demonstration of genome edited trees being produced in a conifer species. This opens the way to apply genome editing for both fundamental science and economically and environmentally important trait improvements. Optimisation of RNP-based protocols, to achieve transgene free delivery, would facilitate rapid deployment where DNA-free editing is not regulated as a GMO.

## Methods

### Vector construction and selection of target sequences

Several different Pol III promoters have been used to produce guide RNAs for genome editing [48, 76, 77]. It has been suggested that endogenous promoters may result in higher gRNA expression than the widely used Arabidopsis or wheat promoters [78–80]. We assumed this may be beneficial and used the sequences of the A. thaliana U6-26 snRNA (NCBI accession no: X52528) [79] and wheat U6 gene (NCBI accession no: X63066) sequences [54] to identify upstream U6 promotor regions in the *Pseudotsuga menziesii* (Mirb.) Franco (Douglas fir) genome (V. 1.0) [81]. We verified the sequence of U6 promoter (NCBI accession no: MW757988) and used this sequence in all single gRNA constructs. For constructs expressing two guide RNAs we used the Douglas fir U6 promoter and the wheat U6 promoter listed above.

All editing targeted the GUX1 gene (NCBI accession no: MT628352) in *P. radiata*. Prior to gDNA design the genomic sequence of GUX1 was recovered from the genotype to be edited. Primers, Primer2 (5` CGATCTCTTGGCTTTTGAGG 3`) and Primer4 (5` TGCCGTGTAGCTTATTGCAG 3` (Fig. 2A), were used to sequence both alleles. For editing, gDNAs site1: 5` GCAGAAGATCACATGAACAGAGG 3`, site2: 5` ATTTAGGGACTCGGAAAGGGGG 3`, site3: 5` GGTTGTATTGCTATCAACGGCGG 3` and site4: 5` AGCTGCAGGTTGGAAACTGCGG 3` were selected from regions without allelic differences (Fig. 2A). To increase the efficiency of genome editing gRNAs were designed according to the protocols of Dang et al. [82]. Additionally, the U6-26 terminator was added to the 3` end of the gRNA structure for double gRNA vectors. We used the online tools E-CRISP [83] and CRISPOR [84] to identify target sites in the gene (Fig. 2A). The target sites were selected based on efficiency and activity scores [85–87]. All vectors were synthesized by GenScript (Piscataway, NJ, USA). Vector maps are provided in Fig. S1 (Additional file 1).

### Plant transformation

#### Plant material

All experiments were carried out using previously cryopreserved somatic embryogenic cell lines of *P. radiata* that were initiated at Scion. These lines were established from immature embryos harvested from green cones and proliferated on Glitz2, a modified Litvay media [88] before being cryopreserved.

#### Tissue preparation

*P. radiata* somatic embryogenic cell lines, recovered from cryopreservation were proliferated by fortnightly lump transfers on a Litvay-based media [89] as modified by Hargreaves et al. (Glitz2) [88] with an additional 2 mg/L glycine. Three weeks prior to bombardment, tissue was suspended in GLITZ0 (Glitz2 liquid media with no plant growth regulators) (1 g fresh weight: 5 ml liquid media). One ml aliquots of the suspension were pipetted onto sterile Whatman #1 filter paper disks and placed onto fresh media. One week prior to bombardment, filter papers containing tissue were transferred to fresh media. Approximately 20 h prior to bombardment, tissue on the filter paper was re-suspended following the protocol described for the first suspension. The tissue in closed petri dishes was left in the laminar flow overnight to allow excess water to evaporate and bombarded the following day.

#### Particle Bombardment using plasmid DNA

Plasmid DNA (2–5 µg) was precipitated onto gold particles (< 10 μm, Sigma Aldrich) by mixing with 50 µl of gold (1 mg/10µl), 20 µl of 0.1 M spermidine and 50 µl of 2.5 M CaCl_2_. After 20 min at room temperature, 90 µl of supernatant was removed and discarded. From the remaining solution, 5 µl aliquots were placed onto six Swinnex filter holders (13 mm, Millipore, USA) for bombardment of the prepared somatic embryogenic tissue with a Particle Inflow Gun [90] using the following settings: helium pressure, 900 kpa; solenoid valve opening time, 30 ms; shooting distance, 19 cm (lowest shelf position); chamber vacuum, -96 kPa (-14 psi). Tissue was then placed in a dark culture room at 24 °C.

#### CRISPR-RNP complex assembly (SpCas9/gRNA 1:1.2 molar ratio)

Custom synthesized crRNAs (10 nmol), tracrRNA (100 nmol), Alt-R SpCas9 Nuclease 3NLS (61 µM) and Alt-R SpCas9 nuclease V3 (61.6 µM) were obtained from Integrated DNA Technologies, (Coralville, IA, USA). The lyophilized crRNAs and tracrRNA stocks were dissolved in nuclease free duplex buffer to obtain a concentration of 100 µM. To form 25 µM gRNA duplexes (site1 or site3); 3 µl of crRNA and 3 µl of tracrRNA were mixed with 6 µl of duplex buffer and annealed by heating to 95^o^C for 5 min and slowly cooling to RT. The RNP complexes were assembled by mixing 6 µl of each gRNA duplex with 2 µl of Alt-R SpCas9 nuclease 3NLS or Alt-R SpCas9 nuclease V3 and 1 µl each of 10x PBS (pH 7.4) and sterile H_2_O. The solution was carefully mixed by pipetting, incubated at RT for 30 min and stored at -20^o^C until required.

#### Particle Bombardment using Hybrid RNP complex/selection vector

To prepare the gold particles 5 µl of each RNP complex (site1 or site3) and 2.5 µl of pRN2; a ZmUbi promoter::NptII geneticin selection vector (1.5 µg/µl) [9] were added to a microfuge tube containing 7 mg gold in 30 µl sterile H_2_O and mixed thoroughly by pipetting. Particle bombardment was performed as above using 7 µl aliquots of coated gold.

#### Post bombardment selection

After 24 h, the filter papers with embryogenic tissue were placed onto selection media (Glitz2 containing 15 mg/L geneticin) and returned to the dark room. Four to eight weeks later putative transgenic tissue was isolated and proliferated on selection media for one month. Confirmed genome-edited embryogenic tissue was proliferated without selection until a sufficient amount of material was available for plant maturation protocols.

#### Somatic embryo maturation and germination

To obtain mature somatic embryos 5 × 100 mg lumps of 10-day old Glitz2-proliferated tissue were transferred onto a Litvay-based maturation medium containing ½ strength Litvay macro nutrients, full strength micro nutrients (with the exception of manganese sulphate at 0.124 mM concentration) [89], supplemented with the iron, vitamins, and amino acids from Smith EDM medium [91], 60 g/L of sucrose, 56.7 µM abscisic acid, and 8 g/L gellan gum (Phytagel®, Sigma). Cultures were grown in the dark at 24^o^C. After two weeks the tissue was subcultured onto fresh maturation media of the same composition and grown for a further 8–12 weeks. Mature somatic embryos were periodically harvested onto a modified BMG-2 [92, 93] germination medium, KNV87 [88], grown at 24 °C for 7 days in the dark, then an additional 7 days in low light (under shade-cloth) before being moved to full light conditions (90 µmol m^− 2^ s^− 1^, 16 h photoperiod). Germinated embryos were moved to a modified Quoirin and Lepoivre medium (LP) [94, 95] containing 5 g activated charcoal (LPch) for plantlet multiplication or elongation, as described by C Hargreaves and M Menzies [96].

### Screening and analysis

For initial screening, DNA was extracted using CTAB [97] from 30 to 50 mg of post bombardment embryogenic tissue growing on selective media and screened for editing by PCR and sequencing. PCR was performed using 50 ng of genomic DNA and Q5 polymerase (NEB) following the manufacturer’s instructions with annealing conditions of 60^o^C for 30 s and using primer1 (5` CCTGAAAACCCTAACCTGCTTC 3`) and primer3 (5` CCAGAGTTGAAGAGTGTTGCAT 3`) (Fig. 2A). PCR products were either gel extracted or purified using exonuclease I and shrimp alkaline phosphatase (Exo-SAP) and sequenced by Sanger sequencing using primer2 and primer3 (Fig. 2A). Sequencing results were analysed by manual screening and the online tools ICE (https://ice.synthego.com/ ) and TIDE (http://shinyapps.datacurators.nl/tide/) [98]. Embryogenic tissue confirmed to harbour GUX1 edits were matured to produce somatic embryos and these were germinated and multiplied. Putatively edited plantlets were then characterized by cloning of PCR products generated from the plantlets into an *E. coli* vector and sequencing of 10 independent clones for each plantlet.

## Supplementary Information

**Additional file 1: Figure S1.** Vector maps of plasmids used in transformation experiments.

**Additional file 2: Figure S2.** Chromatograms of sequencing done on the edited embryogenic tissue and plants.

**Additional file 3: Figure S3.** Electrophoresis gel pictures of PCR from editing with plasmid DNA and RNPs.

**Additional file 4: Table S1.** Summary of somatic embryogenic lines and plants generated.

## Data Availability

The sequence of *PrGUX1* has been deposited in GenBank of NCBI with accession No. of MT628352.1 (https://www.ncbi.nlm.nih.gov/nuccore/MT628352.1/). The sequence of *Pseudotsuga menziesii* (Douglas fir) U6 small nuclear snRNA (U6) gene sequence has been deposited in GenBank of NCBI with accession No. of MW757988.1 (https://www.ncbi.nlm.nih.gov/nuccore/MW757988). All other data generated or analysed during this study are included in this published article.

## Abbreviations

CRISPR: Clustered regularly interspaced short palindromic repeats
ICE: Inference of CRISPR Edits
INDEL: Insertion or deletion
PCR: Polymerase chain reaction
snRNA: Small nuclear RNA
TALEN: Transcription activator-like effector nucleases
TIDE: Tracking of Indels by Decomposition
ZFN: Zinc finger nuclease
