# Supplementary material for: Genome editing with CRISPR/Cas9 in Pinus radiata (D. Don)
Source: BMC Plant Biol. 2021 Aug 10;21:363. doi: 10.1186/s12870-021-03143-x (PMC8353756; doi:10.1186/s12870-021-03143-x)
Supplement: Supplementary file 1 — Additional file 1: Figure S1. Vector maps of plasmids used in transformation experiments. [file 12870_2021_3143_MOESM1_ESM.docx]

1. **Plasmid encoding a Single gRNA and a plant selectable marker**

**
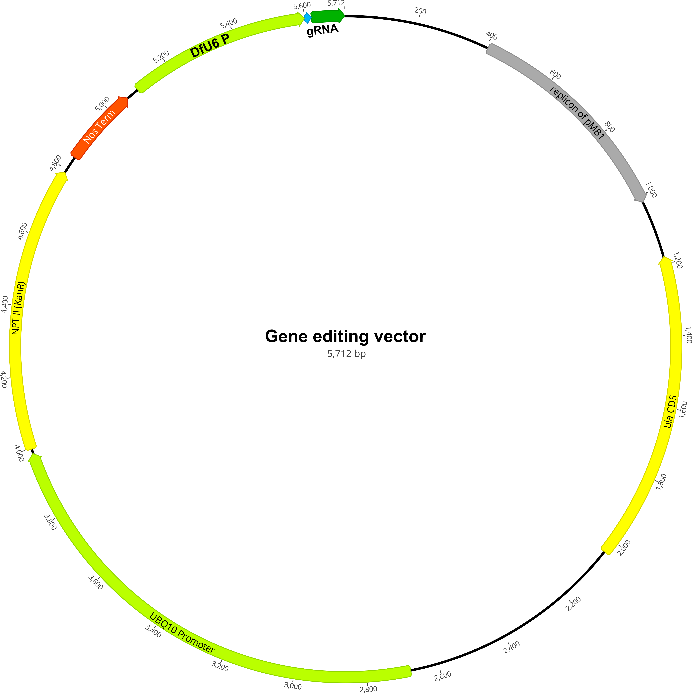
**

1. **Cas9 plasmid for gene editing with a single gRNA**

**
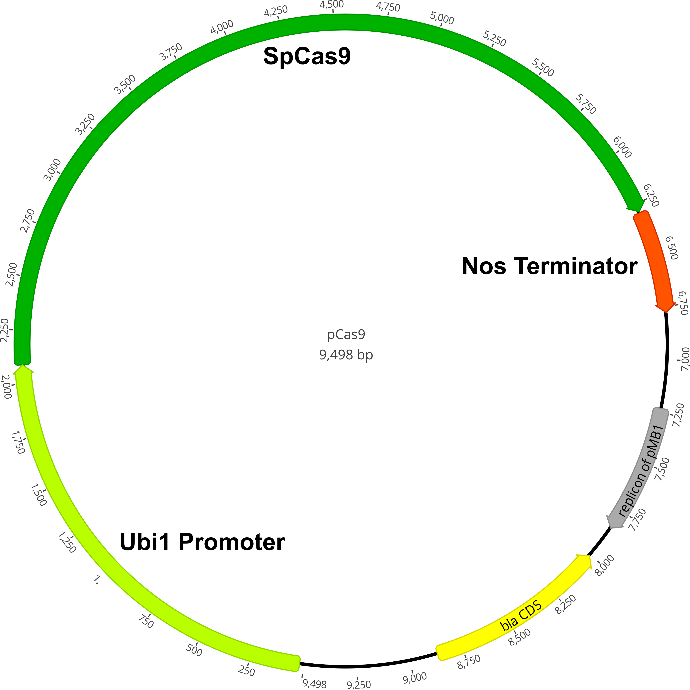
**

1. **Plasmid used for editing with two gRNAs**

**
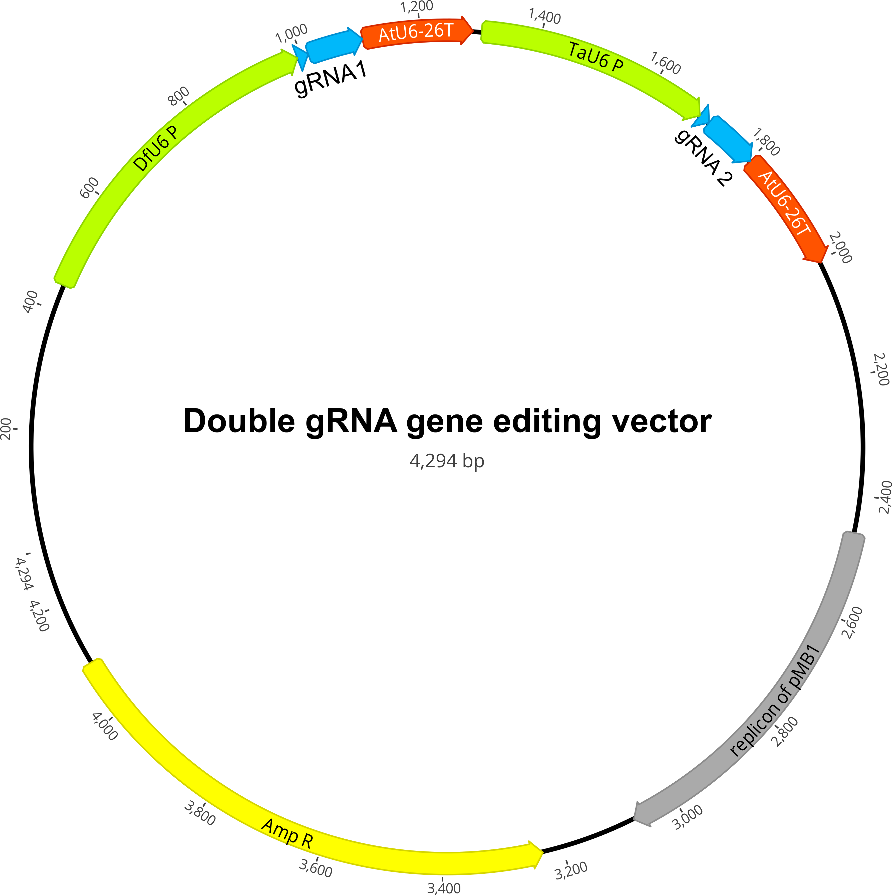
**

1. **Cas9 plasmid with a plant selectable marker used with double gRNA vector**


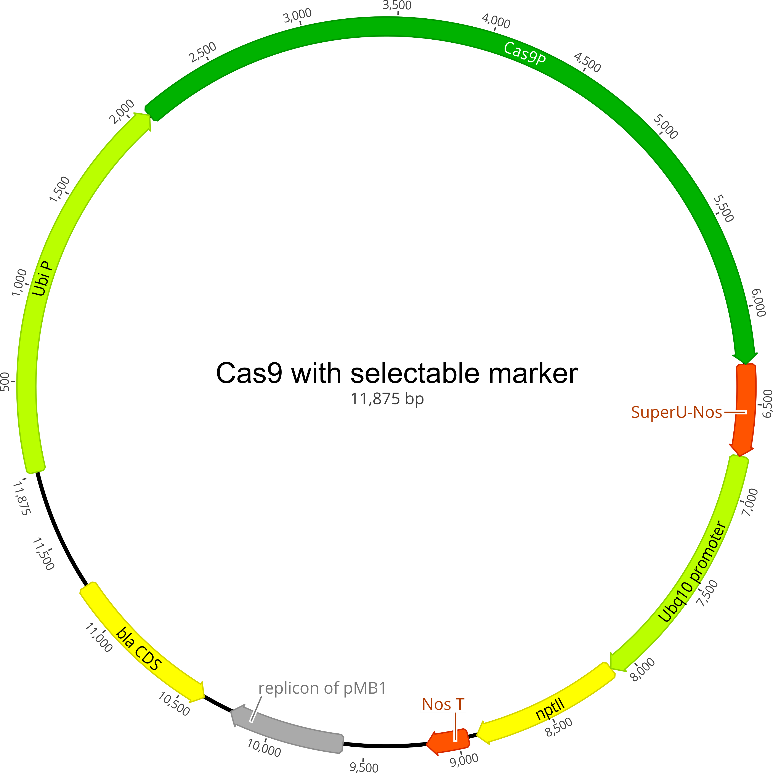


1. Plasmid carrying a plant selectable marker used in RNP experiment


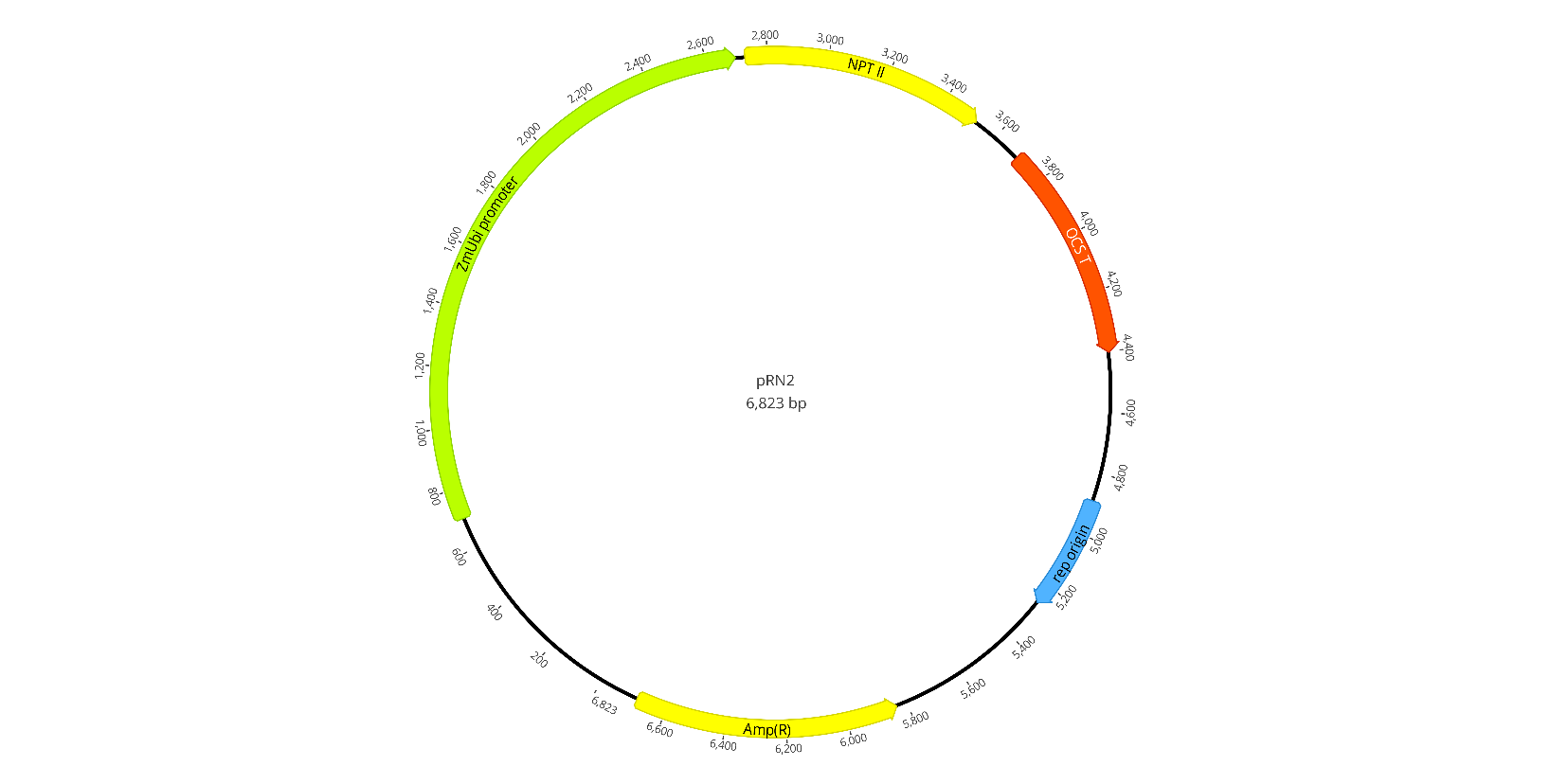


Fig S1: Maps of the vectors used in this study. A. Plasmid containing a single gRNA and a selectable marker for editing a single target site. This plasmid was used as the control plasmid in single gRNA experiments. B Plasmid containing the Cas9 driven by Ubiquitin promoter used in conjunction with the single gRNA vector. C. Plasmid vector containing two gRNA. First gRNA is driven by Douglas fir U6 promoter and the second gRNA by the wheat U6 promoter. This plasmid was used for targeting two target sites. D. Plasmid containing the Cas9 cassette and the selectable marker cassette that was used in conjunction with the plasmid encoding 2 guide RNAs. This plasmid was used as a control vector in two gRNA experiments. E. Plasmid carrying a selectable marker that was used in the hybrid RNP experiments.
