# Supplementary material for: Genome editing with CRISPR/Cas9 in Pinus radiata (D. Don)
Source: BMC Plant Biol. 2021 Aug 10;21:363. doi: 10.1186/s12870-021-03143-x (PMC8353756; doi:10.1186/s12870-021-03143-x)
Supplement: Supplementary file 2 — Additional file 2: Figure S2. Chromatograms of sequencing done on the edited embryogenic tissue and plants. [file 12870_2021_3143_MOESM2_ESM.docx]

1. **Chromatograms of gene edited embryogenic tissues**

**
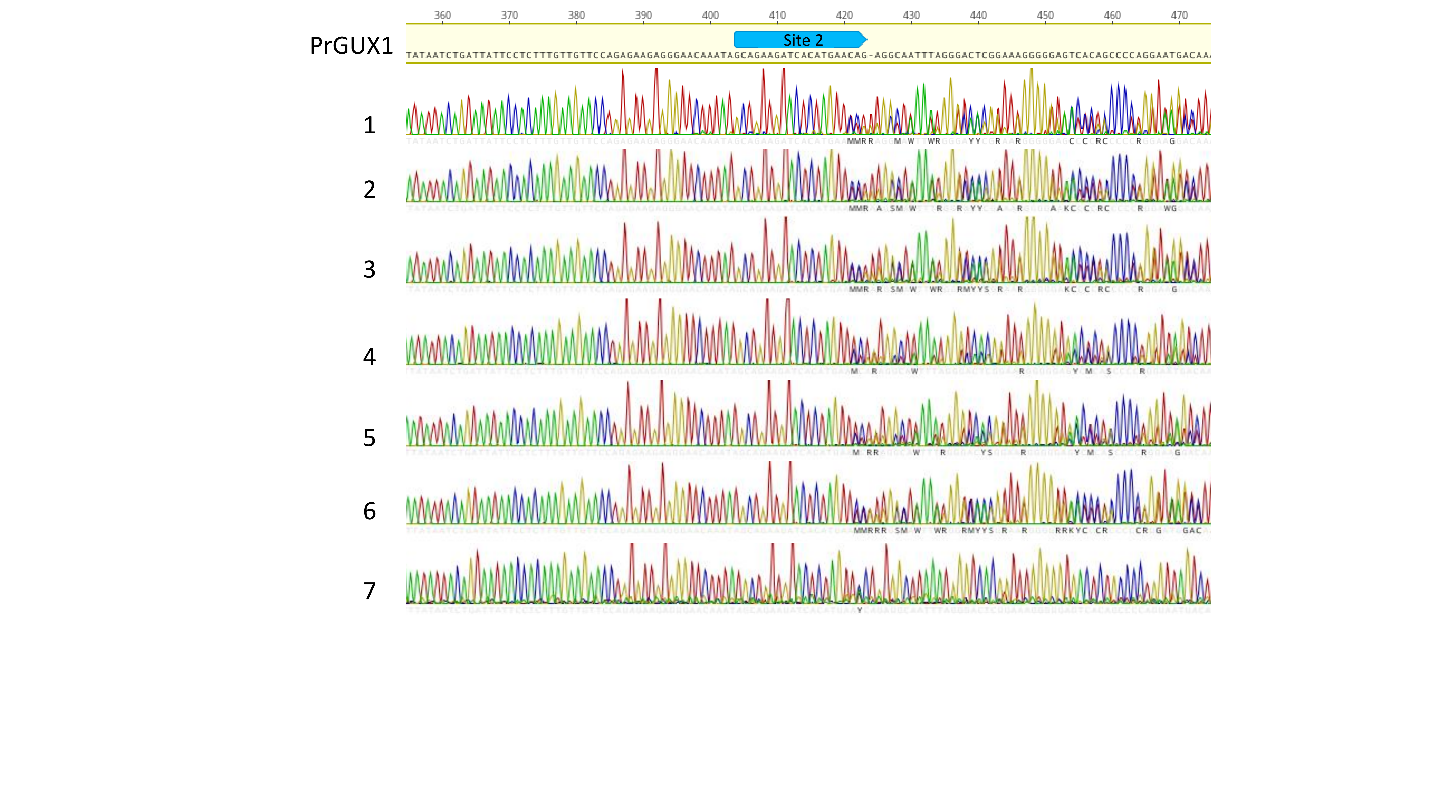
**

1. **Chromatogram of gene edited plants from plasmid DNA experiments**


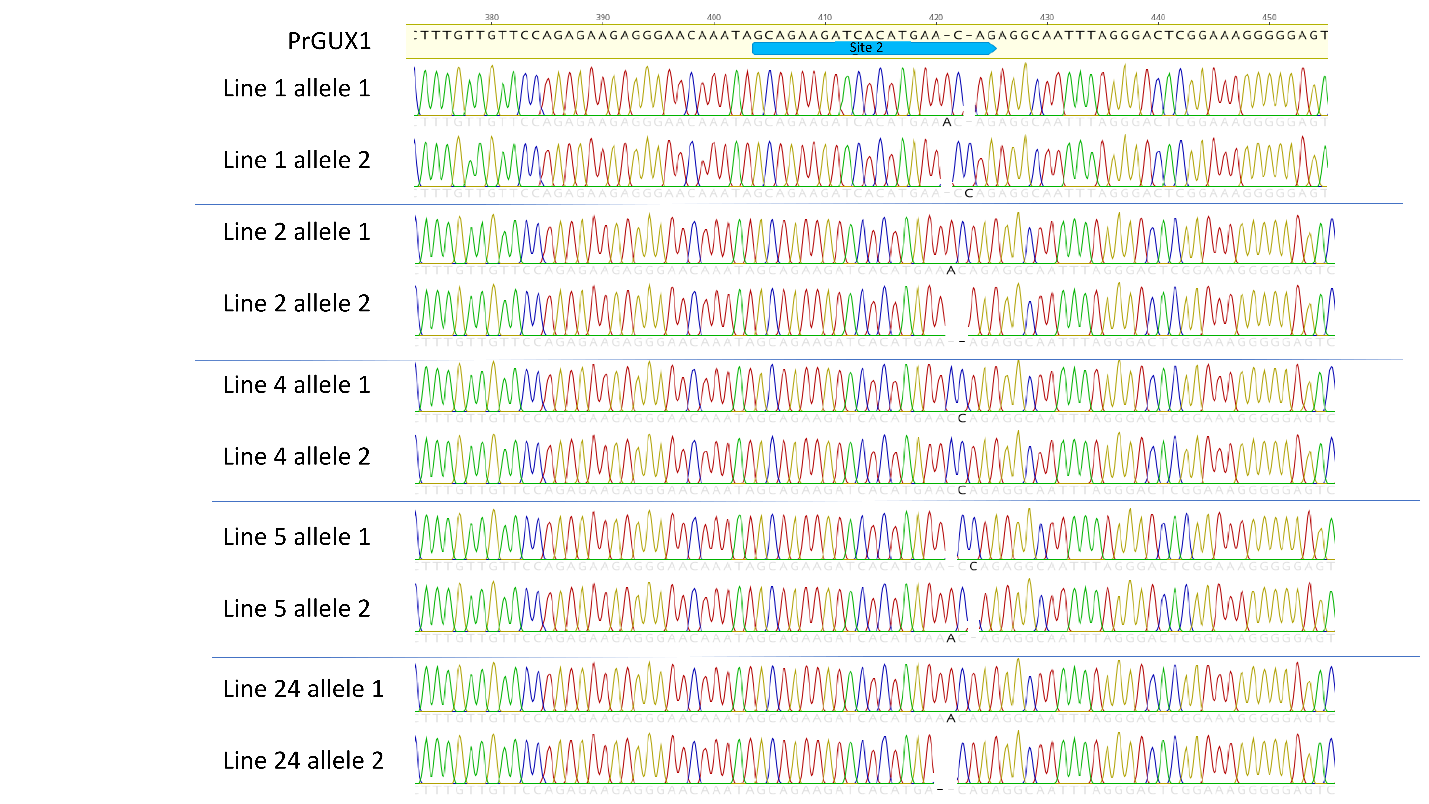


1. **Chromatogram of 1.37kb deletion**


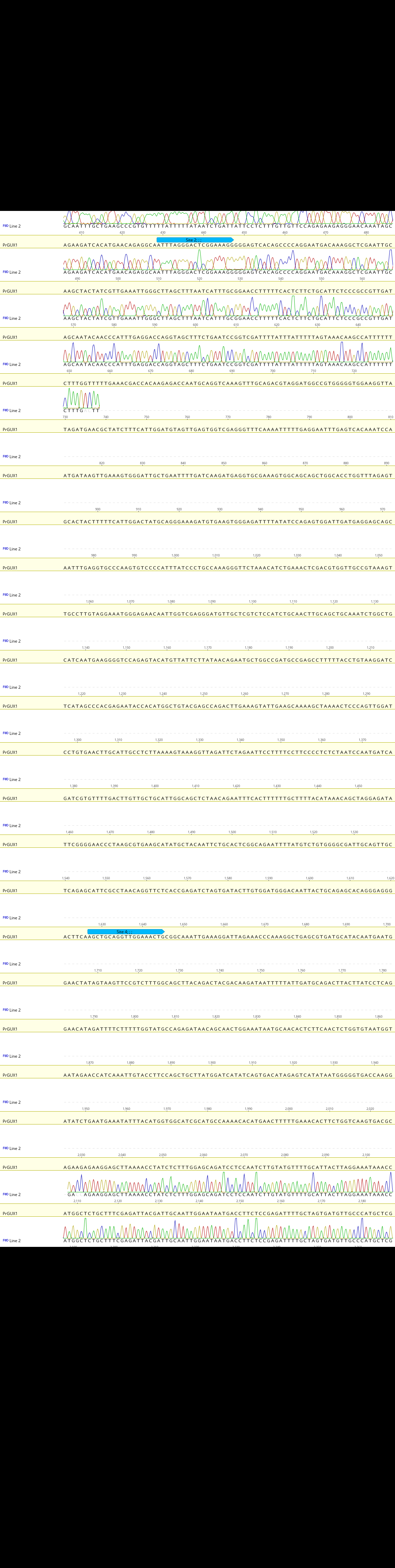


1. **Chromatogram of 1.2kb deletion**


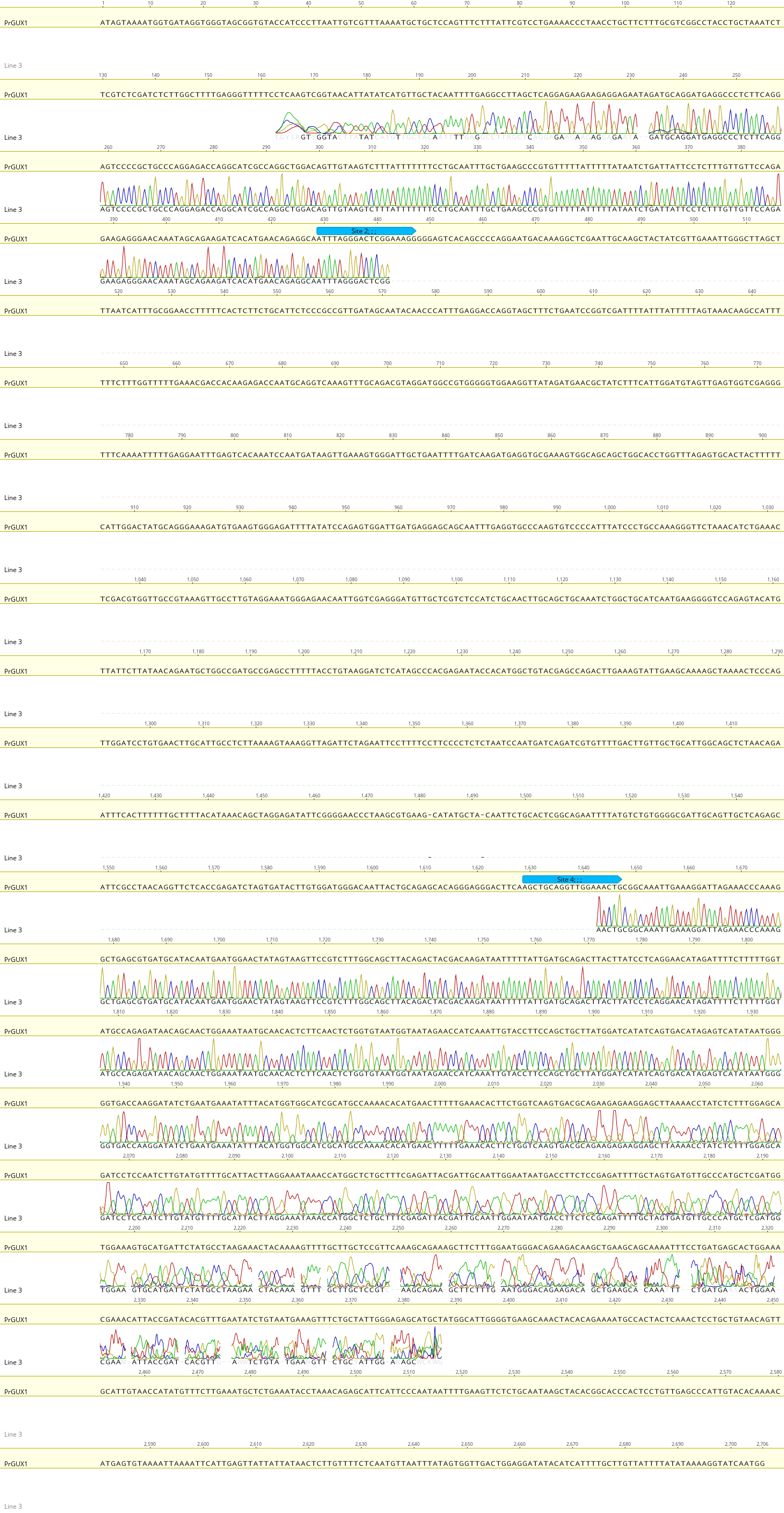


1. **Chromatogram of gene edited plants from hybrid RNP experiments**

**
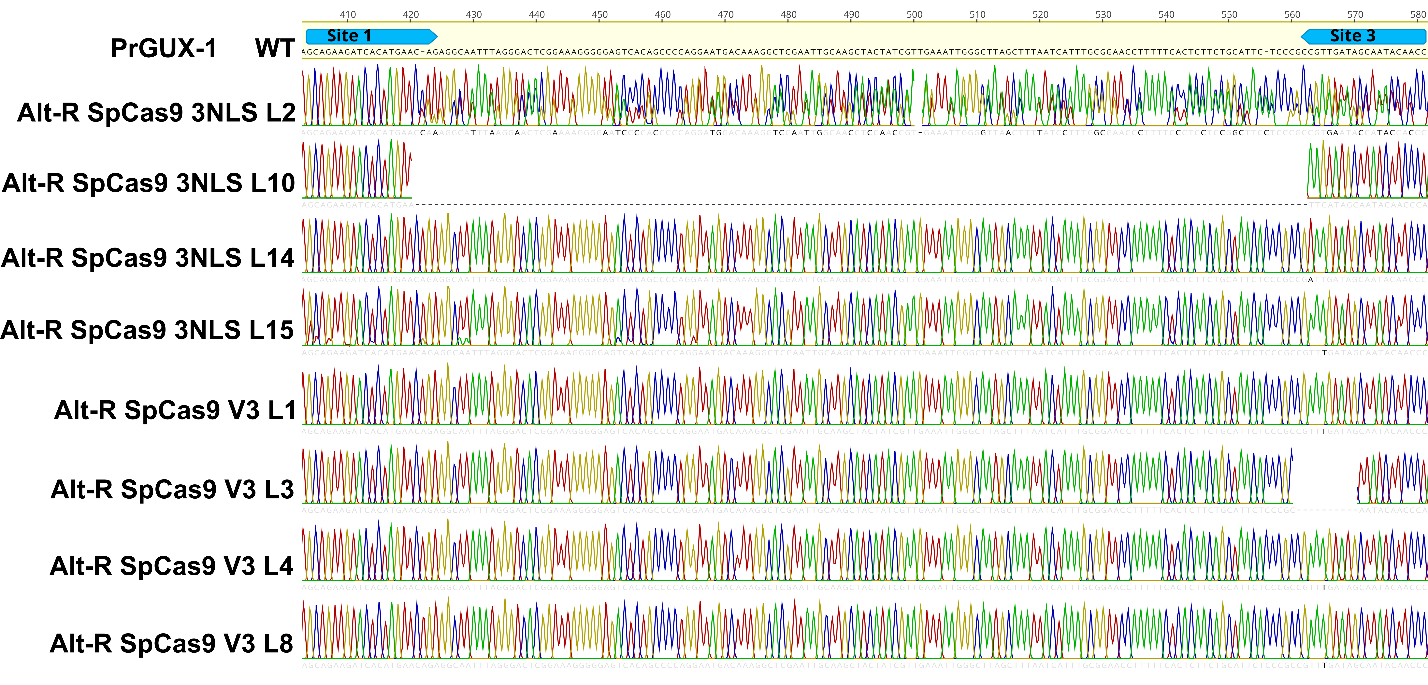
**

**Fig S2. Chromatograms of the CRISPR edited lines. A Chromatograms of gene editing in somatic embryogenic cells with a single gRNA. B Chromatograms of gene editing in plants regenerated from somatic embryogenic tissues with a single gRNA. C Chromatogram of 1.3kb deletion generated by a pair of gRNA in somatic embryogenic cells. D Chromatogram of 1.3kb deletion generated by a pair of gRNA in somatic embryogenic cells. E Chromatogram of gene edited plants from hybrid RNP experiments showing deletions and insertions.**
