# Supplementary material for: Genome editing with CRISPR/Cas9 in Pinus radiata (D. Don)
Source: BMC Plant Biol. 2021 Aug 10;21:363. doi: 10.1186/s12870-021-03143-x (PMC8353756; doi:10.1186/s12870-021-03143-x)
Supplement: Supplementary file 3 — Additional file 3: Figure S3. Electrophoresis gel pictures of PCR from editing with plasmid DNA and RNPs. [file 12870_2021_3143_MOESM3_ESM.docx]

**
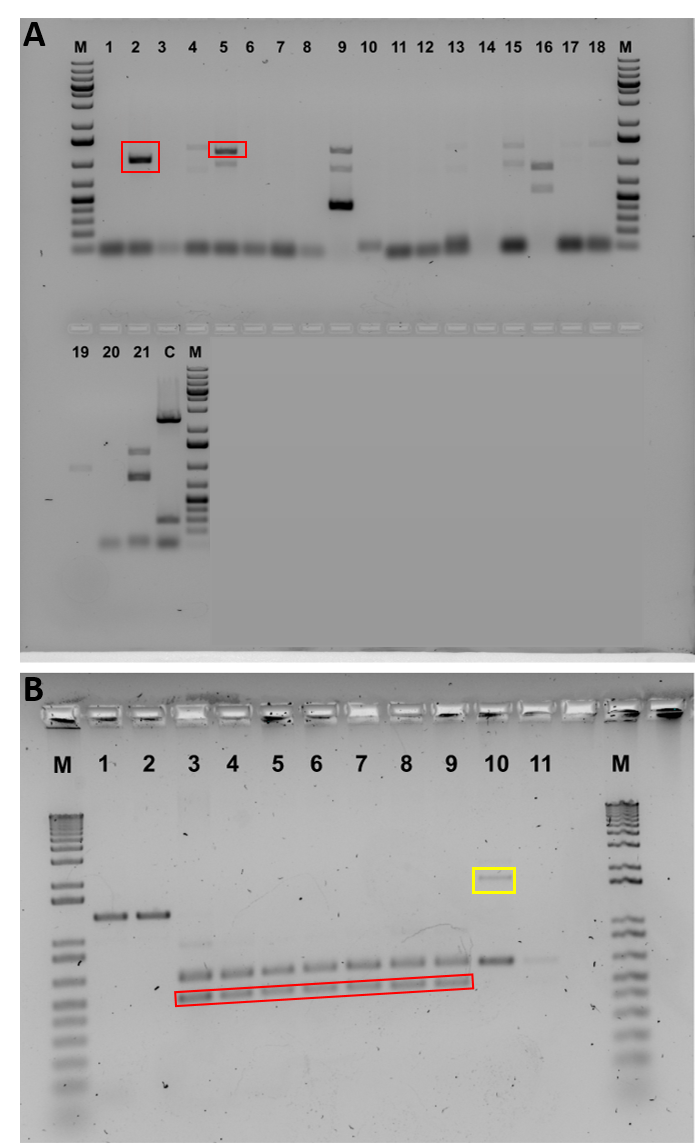
**

**Fig S3.** Electrophoresis gel pictures of PCR from editing with plasmid DNA and RNPs**. A. Electrophoresis gel (1% agarose TAE) picture of gene editing of PrGUX1 in somatic embryogenic tissues using plasmid DNA. Numbers denote lane numbers. Lane M denotes GeneRuler 1kb plus DNA ladder. Lane C denotes PCR band of wild type control plant (2411bp). Red boxes denote the PCR band of sizes 1024bp and 1213bp of lines 2 (lane 2) and 3 (lane 5). Lanes 9 15,16, 19, 21 were nonspecific amplification confirmed by sequencing. B. Electrophoresis gel (1.5% agarose TAE) picture of gene editing of PrGUX1 in somatic embryogenic tissue and plants using RNPs. Lane M denotes Invitrogen™ 1 Kb Plus DNA Ladder. Red boxes indicate the 142 bp deletions in somatic embryo (lane 3) and plants (lanes 4-9) from line Alt-R SpCas9 3NLS L10. Lane 1 & 2 are PCR from the Alt-R SpCas9 V3 L3 and L8 edited plants respectively. Lane 10 is PCR from line Alt-R SpCas9 3NLS L20, yellow box indicates the 1130bp insertion. Lane 11 shows PCR of wild type control plant (628bp).**
