# Supplementary material for: Genome editing with CRISPR/Cas9 in Pinus radiata (D. Don)
Source: BMC Plant Biol. 2021 Aug 10;21:363. doi: 10.1186/s12870-021-03143-x (PMC8353756; doi:10.1186/s12870-021-03143-x)
Supplement: Supplementary file 4 — Additional file 4: Table S1. Summary of somatic embryogenic lines and plants generated. [file 12870_2021_3143_MOESM4_ESM.docx]

Table S1: Summary of somatic embryogenic lines and plants generated

|  | Embryogenic lines generated | Edited lines generated | Surviving lines | Embryos germinated | Edited plantlets tested | Allelic variation |
| --- | --- | --- | --- | --- | --- | --- |
| Editing with single gRNA | 100 | 15 |  |  |  |  |
|  |  |  | Ln1 | 15 | 5 | Biallelic |
|  |  |  | Ln24 | 1 | 1 | Biallelic |
| Editing with double gRNA | 100 | 12 |  |  |  |  |
|  |  |  | Ln2 | 5 | 1 | Biallelic |
|  |  |  | Ln3 | 10 | 4 | Biallelic |
| Editing with RNPs |  |  |  |  |  |  |
| Alt-R SpCas9 3NLS | 22 | 5 |  |  |  |  |
|  |  |  | Ln2 | 20 | 13 | Monoallelic |
|  |  |  | Ln10 | 24 | 3 | Monoallelic |
|  |  |  | Ln14 | 17 | 3 | Monoallelic |
|  |  |  | Ln15 | 16 | 3 | Monoallelic |
|  |  |  |  |  |  |  |
| Alt-R SpCas9 V3 | 12 | 4 | Ln1 | 37 | 3 | Monoallelic |
|  |  |  | Ln3 | 24 | 3 | Monoallelic |
|  |  |  | Ln4 | 17 | 3 | Monoallelic |
|  |  |  | Ln8 | 46 | 3 | Monoallelic |
| Transgenic control | 4 |  |  | 10 | 8 | No edits |
| Non-transgenic control | NA | NA | NA | 15 | 6 | No edits |
